# Photoplethysmography-documented atrial fibrillation in the first week after catheter ablation is associated with lower success rates

**DOI:** 10.3389/fcvm.2023.1199630

**Published:** 2023-06-22

**Authors:** Martin Manninger, Astrid N. L. Hermans, Andrei-Antonio Caracioni, Ursula Rohrer, Anna-Sophie Eberl, Kevin Vernooy, Andreas Zirlik, Dominik Linz, Daniel Scherr

**Affiliations:** ^1^ Division of Cardiology, Department of Internal Medicine, Medical University of Graz, Graz, Austria; ^2^ Department of Cardiology, Cardiovascular Research Institute Maastricht (CARIM), Maastricht University Medical Centre, Maastricht, Netherlands

**Keywords:** atrial fibrillation, atrial fibrillation ablation, blanking period, remote rhythm monitoring, mHealth, photoplethysmography

## Abstract

**Aims:**

To test the feasibility of postprocedural photoplethysmography (PPG) rhythm telemonitoring during the first week after atrial fibrillation (AF) ablation and its predictive value for later AF recurrence.

**Methods:**

PPG rhythm telemonitoring during the first week after the ablation procedure was offered to a total of 382 consecutive patients undergoing AF ablation. Patients were instructed to perform 1 min PPG recordings by a mobile health application 3 times per day and in case of symptoms. Clinicians assessed the PPG tracings via a secured cloud and the information was remotely integrated into the therapeutic pathway via teleconsultation (TeleCheck-AF approach).

**Results:**

119 patients (31%) agreed to perform PPG rhythm telemonitoring after ablation. Patients included in the TeleCheck-AF approach were younger compared to those who declined participation (58 ± 10 vs. 62 ± 10 years, *p* < 0.001). Median follow up duration was 544 (53–883) days. 27% of patients had PPG tracings suggestive of AF in the week following the ablation. In 24% of patients, the integration of PPG rhythm telemonitoring resulted in a remote clinical intervention during teleconsultation. During follow-up of one year, 33% of patients had ECG-documented AF recurrences. PPG recordings suggestive of AF in the week after ablation were predictive of late recurrences (*p* < 0.001).

**Conclusion:**

PPG rhythm telemonitoring during the first week after AF ablation often triggered clinical interventions. Due to its high availability, PPG-based follow-up actively involving patients after AF ablation may close a diagnostic and prognostic gap in the blanking period and increase active patient-involvement.

## Introduction

Atrial fibrillation (AF) ablation is an established treatment option able to decrease AF burden, progression and AF-related complications (1, 2). Traditionally, a two- to three-month blanking period is used before assessing the long-term outcome of AF ablations accounting for potential pro-arrhythmic effects of the ablation procedure (3). However, multiple studies revealed a correlation between early recurrences during the blanking-period and long-term outcomes (4, 5). Until now, implantable loop recorders, ECG mHealth devices or ECG Holter monitoring of variable duration have been used for rhythm monitoring during follow-up in clinical trials, and the feasibility of novel rhythm monitoring technologies, such as photoplethysmography (PPG) in this clinical setting remains unclear (6).

First clinical experience with the PPG technology in this patient population around AF ablation was collected in 40 AF centres within the TeleCheck-AF project and early adopters of this PPG technology saw a great potential for monitoring post-ablation patients (7–10). Additionally, a recent practical guide by the European Heart Rhythm Association (EHRA) on the use of mobile health technologies proposes, that particularly patient populations with already diagnosed AF without the need of ECG confirmation are best suited for using PPG technology for rhythm telemonitoring as an alternative for ECG technology (11). However, data on feasibility and prognostic implications of PPG rhythm telemonitoring directly after AF ablation are absent.

In this pragmatic single-centre observational study, we report on inclusion rates, adherence and motivation using an approach of PPG-based rhythm telemonitoring within the first week after AF ablation and assess its predictive value for later clinical ECG-documented AF recurrences.

## Materials and methods

### Study population

Consecutive patients undergoing AF ablation between June 1st 2020 and December 15th 2021 at the Medical University of Graz were offered the opportunity to perform PPG telemonitoring within the first week after being discharged from hospital.

This study was approved by the local ethics committee and all patients gave informed consent for inclusion in the ablation registry and, if applicable, telemonitoring within the TeleCheck-AF initiative.

### Telemonitoring

Within 1 week before the ablation procedure, patients presented at the outpatient clinic for informed consent and preliminary exams including lab testing, transthoracic echocardiography, transoesophageal echocardiography and/or computed tomography scans. During this outpatient clinic appointment, patients were given the opportunity to perform telemonitoring within the TeleCheck-AF initiative during the first week after being discharged from the hospital.

**Figure 1:**
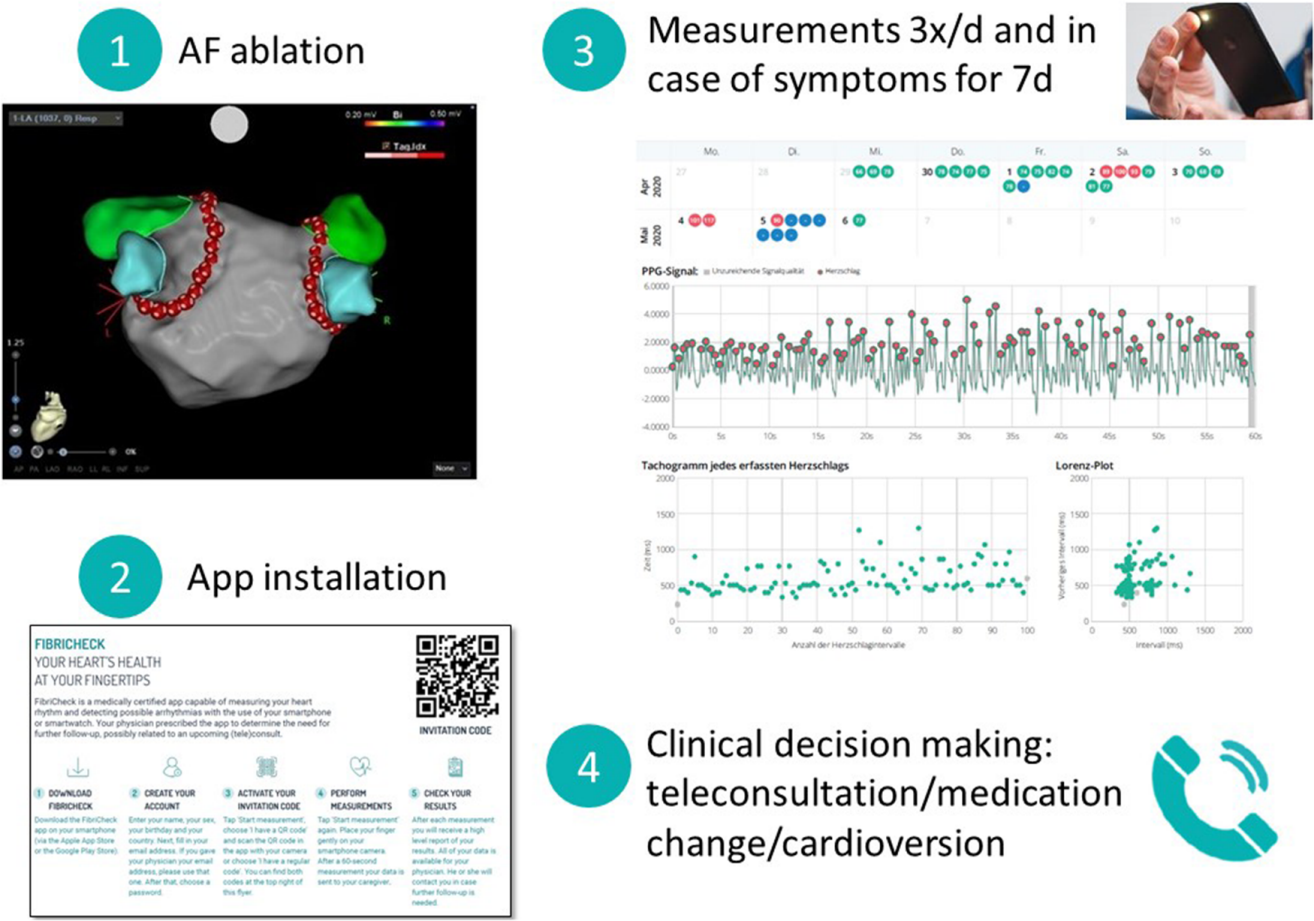
Schematic overview of telemonitoring. AF, atrial fibrillation; d, days.

Patients received an information sheet including a QR code for activation of the FibriCheck® app (Qompium, Hasselt, Belgium) on their smartphone and the study coordinator's telephone number. Patients then either self-installed the app or, upon request, were assisted by study coordinators. After installation of the app, patients were connected to the clinician's telemedicine portal (see schematic Figure 1). Patients were instructed to perform a 1-minute rhythm recording using their smartphone's camera and light source three times per day and in case of symptoms. Patients were included in the study if they installed the app within the first two days after the ablation and performed at least one measurement. Clinicians assessed the tracings via a secured cloud and contacted the patients if therapeutic steps were indicated.

### Motivation and adherence

Motivation was defined as the proportion of days the patient performed at least the required number of measurements (3). Adherence was defined as proportion of performed measurements over the total number of required measurements over a duration of 6 complete days (first and last half days not counted, 3 measurements per day over a duration of 6 days = 18 measurements required, can be >100% if more measurements than necessary were performed).

### Ablation procedure

Ablation procedures were performed on the day of hospital admission. Patients received oral anticoagulation for at least four weeks prior to the procedure. In case of insufficient anticoagulation, transoesophageal echocardiography was performed prior to the procedure to rule out left atrial thrombus. All procedures were performed under conscious (radiofrequency or cryo ablations) or deep sedation (pulsed field ablations) using fentanyl and propofol.

In case of a first procedure, pulmonary vein isolation was either performed with radiofrequency ablation (CARTO 7, ablation catheter: QDOT Micro or Thermocool SmartTouch, mapping catheter: Lasso or PentaRay, Biosense Webster), cryo-ablation (Arctic Front Advance and Achieve catheter, Medtronic) or pulsed field ablation (Farawave 31/35 mm, Boston Scientific). Pulmonary vein isolation was verified with entrance and exit-block pacing of all pulmonary veins.

In case of a repeat procedure, a 3D electroanatomic mapping system was used (CARTO 7, Biosense Webster). After transseptal puncture, a multielectrode mapping catheter (PentaRay, Biosense Webster) was used to map the left atrium. In case of AF, cardioversion was performed before mapping. If cardioversion was unsuccessful, the left atrium was mapped in AF. Gaps in the antral pulmonary vein isolation were closed using radiofrequency ablation (Thermocool SmartTouch, Biosense Webster).

Additional ablation of the cavotricuspid isthmus was performed in case of typical right atrial flutter documented either prior to or during the procedure. Further ablation was performed in case of persistent AF at the operator's discretion.

Anticoagulation was restarted on the day of the procedure for at least three months after the ablation and was continued thereafter according to the patients' risk profile.

In case of previously prescribed antiarrhythmic drug therapy, it was continued for three months after the procedure.

### Follow-up

Patients were followed up with 24 h Holter monitoring at our institution or their referring physicians 3, 6 and 12 months after the procedure. After the 12-month follow-up visit, patients were followed up with a yearly 24 h Holter ECG at their referring physician. Between regular visits, patients were encouraged to seek ECG or Holter monitoring in case of symptoms suggesting AF recurrences.

### Data processing and statistical analyses

Continuous variables are presented as mean ± SD, median (range). Categorical variables are presented as percentages (%) and counts. Two-group comparisons of continuous variables were performed by Student's t tests if normally distributed or with Wilcoxon ranksum tests if the normality assumption was violated according to Shapiro-Wilk tests or visual inspection of normal probability plots. Categorical variables were compared by Chi-square tests or Fisher's exact tests. Time to first arrhythmia recurrence was calculated without a blanking period and plotted using the Kaplan-Meier product-limit method with comparisons performed by logrank statistics. Two-tailed *p* values < 0.05 were considered to indicate statistical significance.

Baseline characteristics were complete in all patients. Statistical analyses were performed using SPSS 23.0 (IBM, Armonk, NY).

## Results

Out of 382 consecutive patients undergoing AF ablation between June 1st 2020 and December 31st 2021, 119 patients (31%) performed telemonitoring after ablation. Patients undergoing telemonitoring were younger compared to those who declined (58 ± 10years vs. 62 ± 10years, *p* = 0.001, Table 1). CHA_2_DS_2_-VASc scores, gender, types of AF, presence of atrial flutter and number of previous ablations were comparable between both groups.

**Table 1:** Baseline characteristics of patients with atrial fibrillation (AF) performing vs. patients declining photoplethysmography (PPG) rhythm telemonitoring.

	No PPG monitoring (*n* = 263)	PPG monitoring (*n* = 119)	*P*
Age (years)	62 ± 10	58 ± 10	**0.001**
Females	34%	33%	0.9
CHA_2_DS_2_-VASc	2 (0–9)	1 (0–6)	0.03
AF type			
– Paroxysmal	67%	62%	0.28
– Persistent	30%	37%	
– Longstanding persistent	6%	1%	
Typical atrial flutter (%)	30%	32%	0.64
Prior ablations	0 (0–3)	0 (0–3)	0.34

Bold values denote significance level *P* < 0.01.

### Patient characteristics (telemonitoring group)

Thirty-four percent of included patients were female, median CHA_2_DS_2_-VASc-Score was 1 (0–6). 62% of patients had paroxysmal AF, 37% had persistent AF and 1% had longstanding persistent AF. One out of four patients (24%) had already undergone previous AF ablations. Most patients (89%) underwent radiofrequency ablations, 7% underwent cryo-ablation and 4% pulsed field ablation. Median follow up duration was 544 (53–883) days. One patient died 53 days after the ablation due to cerebral haemorrhage associated with a direct anticoagulant the patient already received for more than one year prior to the ablation.

### Adherence to PPG rhythm telemonitoring during one week after AF ablation and resulting clinical interventions

Of those patients downloading and activating the PPG app, the median motivation was 33.3% and the median adherence was 77.8%. Motivation and adherence were higher in patients with PPG tracings of atrial tachyarrhythmias in comparison to patients without atrial tachyarrhythmias, 66.6% vs. 33.3% (*p* < 0.05) and 105.6 vs. 78% (*p* < 0.05), respectively.

**Figure 2:**
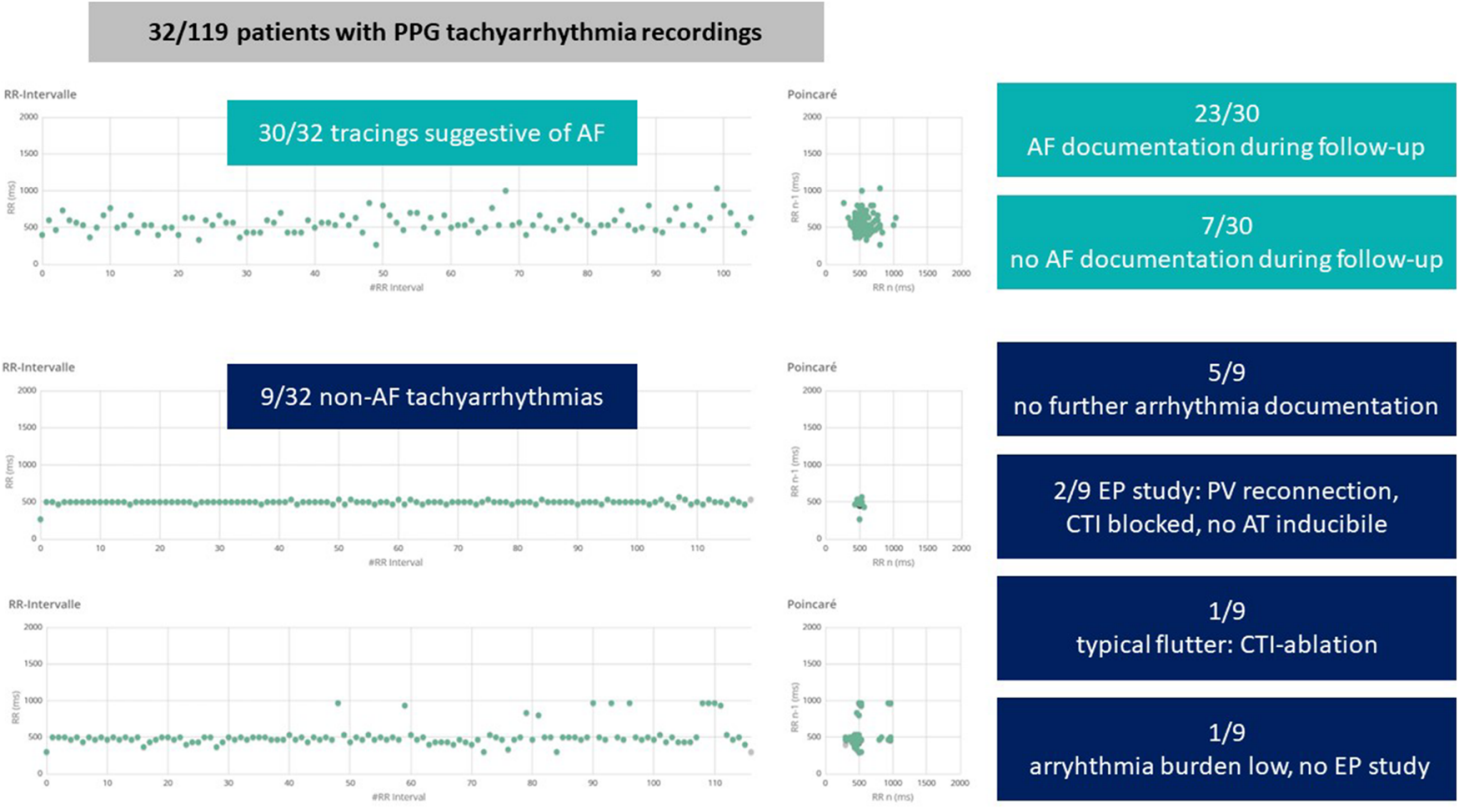
Arrhythmias detected by photoplethysmography (PPG) monitoring during the first week after atrial fibrillation (AF) ablation. Example tachograms (x-axis: time, y-axis: RR interval in ms) and poincaré plots of a tracing suggestive of AF (top), a tracing of a regular tachyarrhythmia (middle) and a tracing suggestive of atrial flutter or atrial tachycardia (bottom). CTI, cavotricuspid isthmus; AT, atrial tachycardia; EP, electrophysiologic.

32 patients (27%) recorded a median of 5 (1–58) tachycardia tracings in the week following the ablation (Figure 2). 30 patients had recordings suggestive of AF, 7 patients had recordings suggestive of AF as well as non-AF tachyarrhythmias and 2 patients recorded only non-AF tachyarrhythmia tracings suggestive of atrial tachycardia or atrial flutter. Complete Holter follow-up (at 3/6/12 months after ablation) was available in 89.9% of patients without documented arrhythmia recurrence.

**Figure 3:**
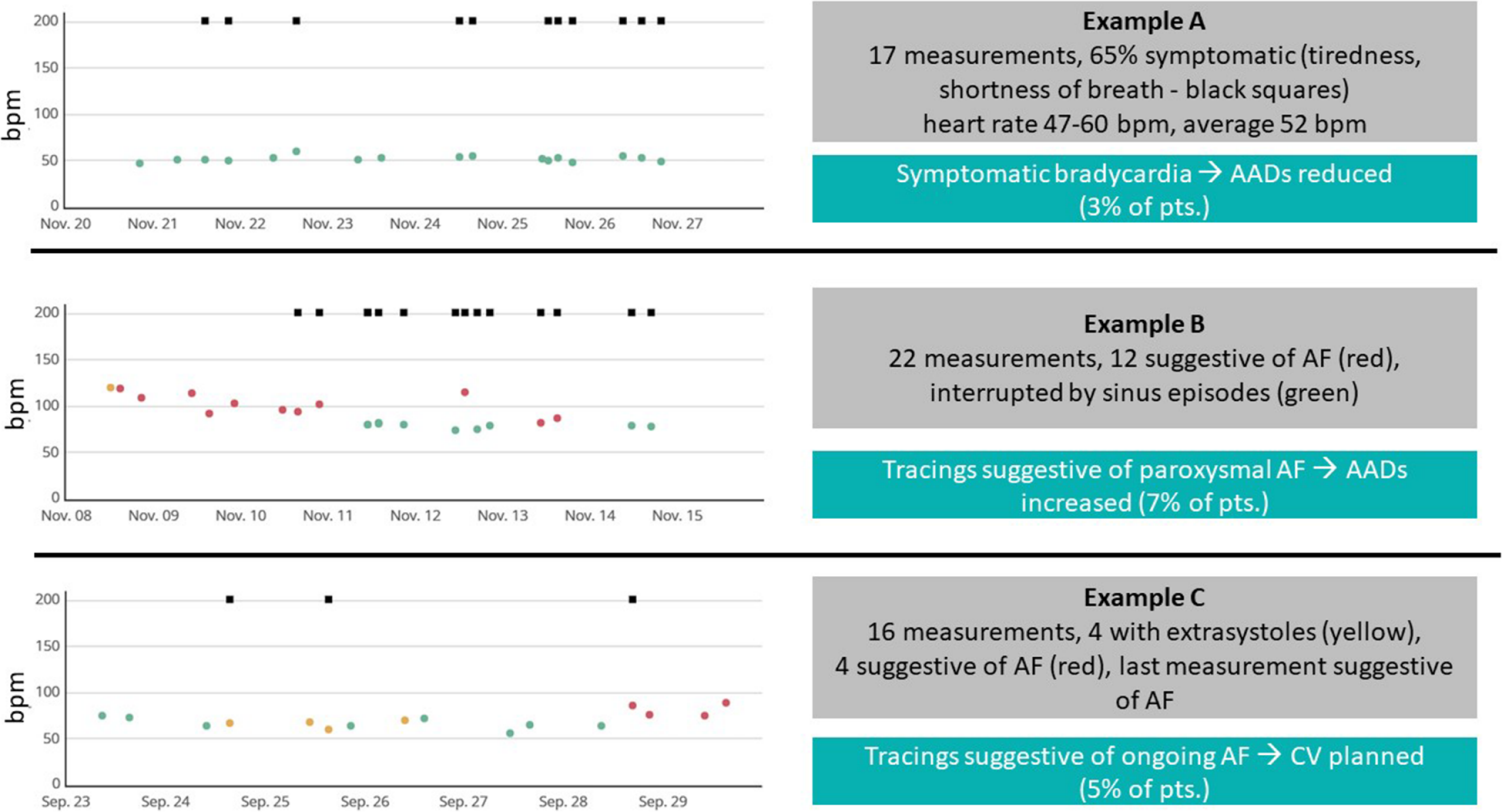
Sample tracings and clinical interventions. Examples of 7-day recordings during the PPG monitoring period. Each point marks one recording (green: regular rhythm, yellow: warning, red: suggestive of atrial fibrillation—AF), squares at the top of each recording mark, whether the patient was symptomatic during the recording. bpm, beats per minute; AAD, antiarrhythmic drug.

Telemonitoring triggered clinical interventions in 24% of patients (*n* = 29, Figure 3): amiodarone was started in 8% (*n* = 10), class I antiarrhythmic drugs were up titrated in 7% (*n* = 8), electrical cardioversion was scheduled in 5% (*n* = 6), antiarrhythmic drugs were reduced due to symptomatic bradycardia in 3% of patients (*n* = 4).

### Association between early PPG-documented and ECG-documented AF recurrence after AF ablation

**Figure 4:**
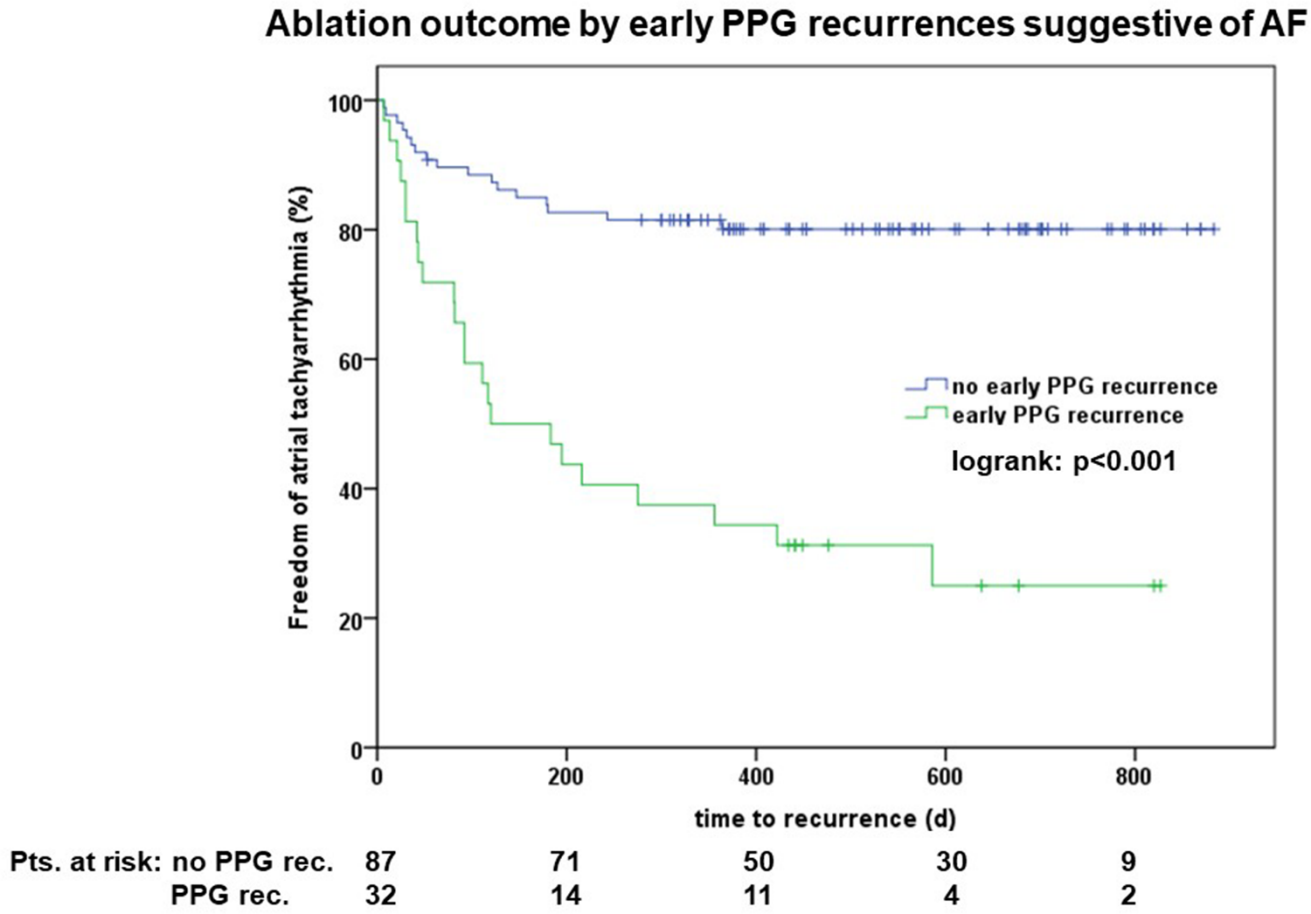
Outcome of patients with vs. without early photoplethysmography (PPG) documented arrhythmias suggestive of atrial fibrillation (AF).

During follow-up, 40 (34%) patients had ECG-documented AF recurrences after a median time of 146 (7–564) days. Twenty percent of the recurrences (*n* = 8) were documented on regular follow-up Holters, 80% (*n* = 32) were documented during patient-initiated ECG recordings. PPG recordings suggestive of AF in the week after ablation were predictive of late recurrences (*p* < 0.001, Figure 4).

The majority of patients neither had early PPGs suggestive of AF, nor late ECG documented AF recurrences (*n* = 70, 59%), 19% (*n* = 23) had both PPGs suggestive of AF and late ECG documented AF recurrences, 14% (*n* = 17) had just late ECG documented AF recurrences and only 8% (*n* = 9) had early PPG AF recurrences during the initial blanking period without later AF recurrences. Sensitivity and specificity of early PPG AF recordings as predictors of late ECG documented AF recurrence were 65.4 and 83.0%, respectively. The positive predictive value was 89.7%, the negative predictive value was 71.9%.

Most of patients (5 out of 9 patients) with early non-AF tachyarrhythmias PPG recordings had no ECG documentations of AF or any other atrial tachyarrhythmia during follow-up (Figure 3). One patient with ECG-documented symptomatic AF recurrence underwent a second AF ablation procedure showing reconnection of pulmonary veins as well as inducible typical atrial flutter and underwent re-do PVI and CTI ablation. Two patients with non-AF tachyarrhythmias underwent repeat procedures showing pulmonary vein reconnection but blocked CTI lines, no atrial tachyarrhythmias were inducible in these patients. One patient refused a repeat procedure because of a significantly reduced arrhythmia burden.

Most patients with ECG documented recurrences underwent redo-ablations (*n* = 17, 43%) or were scheduled for redo-ablations (*n* = 8, 20%). Rhythm control strategy was abandoned in 10% of patients; they were either switched to medical rate control therapy (*n* = 3) or underwent pace and ablate procedure (*n* = 1). Nine patients (22.5%) refused repeat ablations because their symptomatic AF burden was significantly lower and in only two patients (5%) episodes were only documented within the traditional blanking period of three months and required no further intervention.

## Discussion

Holter ECGs are commonly used for follow-up of patients after AF ablation. The use of novel rhythm monitoring devices may overcome limitations of serial ECGs in this clinical scenario. However, concerns have been raised whether these devices are useful in the post-ablation setting, since they were not validated within this patient population which is prone to develop atrial tachyarrhythmias other than AF (11). Here, we demonstrate that intermittent PPG rhythm telemonitoring within the first week after AF ablation using a pragmatic onboarding approach has the potential to close a diagnostic gap during follow-up. Within our patient cohort, an approach of pragmatic PPG rhythm telemonitoring often led to clinical interventions. Most importantly, early PPG-documented AF recurrences within the first week after AF ablation were closely associated with the clinically established ECG-documented long-term rhythm outcomes.

Early arrhythmia recurrences are attributed to atrial and pericardial inflammatory changes induced by the ablation procedure. Traditionally, a 90-day blanking period is used until antiarrhythmic effects of the ablation-induced myocardial scarring take effect (3). This blanking period is commonly used in clinical studies investigating long-term effects of catheter ablation (12–15). However, there is an increasing number of studies highlighting the correlation between recurrences during the blanking period and recurrences after the blanking period while a meta-analysis suggests an optimal blanking period of 4 weeks (4, 5, 16). Importantly, follow-up strategies have been significantly different between the included studies. While multiple studies use implanted cardiac devices to monitor patients after ablation (12), conventional follow-up approaches including repetitive ECGs, Holter recordings and symptom-driven rhythm monitoring are most commonly used in patients outside of clinical trials. We could demonstrate that early PPG-documented recurrences during the first week after ablation were highly predictive of the freedom of atrial tachyarrhythmias after a median follow-up of approximately 1.5 years. This is in line with other trials, meta-analyses and physician-based surveys questioning the benign nature of early atrial tachyarrhythmias recurrences after ablation (17–19).

Novel rhythm monitoring devices might help switching follow-up strategies to a more patient-centred approach allowing low-threshold, long-duration monitoring enabling symptom-rhythm correlation. For example, a prior study has shown that 2 weeks of intermittent monitoring using single-lead ECG devices was superior in detecting AF recurrences and resulted in higher patient convenience than short continuous Holter monitoring (20). Another study demonstrated how single-lead ECG monitoring can be implemented into follow-up of these patients with AF detection rates comparable to standard clinical follow-up (21). PPG monitoring has not been assessed within this patient cohort, but the fact that it requires no specific hardware, but uses the patient's smartphone, is promising for application in everyday clinical practice outside of clinical trials (22). Due to limited ambulatory capacities during the COVID-19 pandemic, several European centres collected experience on using on-demand digital devices for follow-up of patients after ablation (7). Of note, the total inclusion rate of 31% in this single centre study was relatively low in our series of consecutive patients and younger patients were more willing to participate in the study. These two observations differ from the overall results of the complete TeleCheck-AF analysis (23). This may be attributed to the pragmatic onboarding approach or clinical scenario specific factors including general scepticism towards the technology, the timepoint of inclusion during the long preparatory outpatient visit prior to ablation, or limited digital literacy. Additionally, motivation and adherence to perform the recommended number of measurements was lower than in the total TeleCheck-AF cohort (23). However, the higher motivation and adherence in those patients who recorded arrhythmias might reflect the importance of the symptom-driven recordings in this specific clinical scenario post PVI. Personal assistance during the installation process and close monitoring of adherence including measurement reminders might enhance patient acceptance, adherence, and motivation.

One potential limitation of the PPG rhythm telemonitoring is the detection of non-AF tachyarrhythmias. We previously described a structured stepwise approach on how to deal with specific PPG tracings which highlights combining specific tachogram and poincaré plot patterns with the patient's history to choose which further diagnostics steps to take (24). In this study, the prevalence of non-AF tachyarrhythmias was low and most of the documented arrhythmias were only documented within the first week after ablation. There was only one single patient with detection of a previously non-documented clinically relevant arrhythmia (typical right atrial flutter). If, however, tracings suggestive of AF were recorded, these were highly predictive of future AF recurrences, which is also in line with a sub-study of the CIRCA-DOSE trial (18).

While AF ablation procedures have become more standardized, safe, and reproducible within the past years, days spent in hospital have decreased within most centres. Same-day discharge has shown to be feasible and safe with the use of standardized protocols (25). However, early paroxysmal and persistent recurrences as well as side effects of antiarrhythmic drugs might develop within the first days after hospital discharge. On-demand remote monitoring using novel rhythm monitoring devices might facilitate patient-involvement as well as interaction between the patient and health care providers. Indeed, clinical interventions were performed in 1 of 4 patients based on PPG recordings. These interventions included medication changes as well as scheduling for early cardioversions. This type of monitoring might reduce the time to intervention, increase patient satisfaction and positively influence patient outcomes.

In summary, we believe that future applications of PPG monitoring could be (1) monitoring early after discharge, (2) patient-initiated monitoring in case of symptoms and (3) structured periodic monitoring.

## Limitations

The current study describes results from a single centre and included only a limited number of patients in different stages of AF undergoing different ablation strategies. Therefore, results may not be generalizable to all AF patients and ablation centres. The study design relies on accurate recordkeeping and may include bias; therefore, these findings need to be confirmed in a larger randomized controlled trial.

Episodes recorded during PPG monitoring were not validated by simultaneous ECG. However, ECG validation might not be necessary in this patient population with diagnosed AF. This is underlined by the fact that unvalidated PPG tracings suggestive of AF were predictive of later ECG documented AF recurrences.

Despite extensive efforts to detect asymptomatic AF/AT recurrences, true recurrence rates may have been underestimated by a lack of continuous AF monitoring. Predictive value of PPG might have been overestimated by patients from this cohort seeking for ECG documentation more thoroughly.

## Conclusion

A pragmatic approach of PPG rhythm telemonitoring during the first week after AF ablation often triggered clinical interventions in patients actively involved in monitoring. In this cohort, PPG recordings suggestive of AF in the week after ablation were predictive of late ECG-documented recurrences, while recurrence of non-AF PPG documented episodes was rare. Due to its high availability, a structured, PPG-based follow-up actively involving patients after AF ablation may close a diagnostic and prognostic gap and increase active patient-involvement.

## Data Availability

The raw data supporting the conclusions of this article will be made available by the authors, without undue reservation.
